# Evidence That *HFE* H63D Variant Is a Potential Disease Modifier in Cluster Headache

**DOI:** 10.1007/s12031-021-01913-8

**Published:** 2021-09-27

**Authors:** Maria Papasavva, Michail Vikelis, Martha-Spyridoula Katsarou, Vasileios Siokas, Emmanouil Dermitzakis, Christoforos Papademetriou, Konstantinos Karakostis, George Lazopoulos, Efthimios Dardiotis, Nikolaos Drakoulis

**Affiliations:** 1 Research Group of Clinical Pharmacology and Pharmacogenomics, Faculty of Pharmacy, School of Health Sciences, National and Kapodistrian University of Athens, Panepistimiopolis Zografou, 15771 Athens, Greece; 2 Headache Clinic, Mediterraneo Hospital, Glyfada, Greece; 3 Department of Neurology, Laboratory of Neurogenetics, University Hospital of Larissa, Greece, Faculty of Medicine, School of Health Sciences, University of Thessaly, Larissa, Greece; 4 Neurologist, Thessaloniki, Greece; 5 iDNA Genomics Laboratory, Athens, Greece; 6 Department of Cardiothoracic Surgery, University General Hospital of Heraklion, Medical School, University of Crete, 71003 Heraklion, Greece

**Keywords:** Primary headache disorders, Genetics, Iron homeostasis, rs1799945, H63D, Southeastern European Caucasians

## Abstract

Cluster headache (CH) is a primary headache disorder with a complex genetic background. Several studies indicate a potential link between iron homeostasis and the pathophysiology of primary headaches. The *HFE* gene encodes for a protein involved in iron metabolism, while genetic variants in *HFE* have been associated with hereditary hemochromatosis (HH), an iron overload disorder. The objective of the current study was to examine the association of the more common *HFE* H63D variant, with the susceptibility to develop CH and diverse clinical phenotypes in a population of Southeastern European Caucasian (SEC) origin. Genomic DNA samples from 128 CH patients and 294 neurologically healthy controls were genotyped for the *HFE* rs1799945 (H63D) variant. H63D genotypic and allelic frequency distribution did not differ significantly between patients and controls (*p* > 0.05). Subgroup analysis revealed a significantly more frequent occurrence of the variant G allele in chronic compared to episodic CH patients, indicative for a possible correlation of the *HFE* gene with the susceptibility for disease chronification. Although homozygosity for the less prevalent H63D variant G allele was minimal in the CH cohort, the results of the present study are in accordance with previous studies in CH and migraine patients, suggesting that *HFE* H63D variant modifies the disease clinical characteristics. Hence, despite the absence of a per se association with CH susceptibility in the current SEC cohort, variability in *HFE* gene may be potentially regarded as a disease modifier genetic factor in CH.

## Introduction

Cluster headache (CH) is a debilitating primary headache disorder, classified in the group of trigeminal autonomic cephalalgias (TACs) and affecting around 0.1–0.4% of the general population (Snoer et al. 2018). The pain attacks in CH are described as excruciating, strictly unilateral, lasting between 15 and 180 min and usually occurring in bouts up to 8 times daily with a circadian and circannual periodicity (Láinez and Guillamón 2017; Liampas et al. 2020). Ipsilateral, prominent, and consistent cranial autonomic symptoms are accompanying CH attacks (Vikelis and Rapoport 2016; Goadsby 2018). According to the criteria developed by International Classification of Headache Disorders 3rd edition (ICHD-3), CH can be sub-diagnosed as episodic (eCH) or chronic (cCH). In a small proportion of patients (about 10%), CH attacks are characterized as chronic, occurring in bouts lasting one year or more without remission or with pain-free periods lasting less than 3 months (Headache Classification Committee of the International Headache Society (IHS) 2018).

Although CH is considered a neurovascular disorder with hypothalamic activation and trigeminal-autonomic reflex playing a predominant role in disease manifestation, its complex pathophysiology is still partially understood. Population, family, and twin studies support a heritable component for this primary headache, with increased risk for developing CH in first- and second- degree relatives compared to the general population and higher concordance in monozygotic twins (Russell 2004; Gibson et al. 2019); thus, potential genetic risk factors seem to predispose to CH development (O’connor et al. 2020; Waung et al. 2020). Consequently, many studies endeavored to discover the genetic components implicated in the etiopathology of CH. Most genetic association studies investigated variants in candidate genes inferred to be involved in the observed CH pathophysiology-related molecular pathways and/or associated with other primary headache disorders, particularly migraine (Sjöstrand et al. 2002; Pinessi et al. 2005; Schürks 2010; Zarrilli et al. 2015; Ran et al. 2017, 2020; Fan et al. 2018; Fourier et al. 2019; Gibson et al. 2019; Papasavva et al. 2020).

The most frequent type of hereditary hemochromatosis (HH), an endocrine disorder of iron overload, is associated with mutations in the *HFE* gene. The *HFE* (high Fe) gene, located in chromosome 6 (6p21.3), encodes for an atypical major histocompatibility complex (MHC) class Itype glycoprotein (Nixon et al. 2018; Pantopoulos 2018). HFE protein is implicated in iron homeostasis by interacting with transferrin receptor 1 (TfR1) on cellular membranes and thereby negatively regulates transferrin-mediated iron uptake. HFE interacts also with TfR2 and probably additional protein molecules, such as hemojuvelin (HJV) and bone morphogenetic proteins (BMPs) and their receptor (BMPR) via the BMP/small mothers against decapentaplegic (SMAD) signaling pathway, shaping a putative iron-sensing complex that upregulates hepcidin expression, the major negative regulatory hormone of systemic iron homeostasis (Kent et al. 2015; Wu et al. 2015; Jiang et al. 2017; Traeger et al. 2018; Katsarou et al. 2019). Excessive iron stores, elevated plasma iron levels, and inflammation induce hepcidin synthesis whereas is suppressed by hypoxia and erythropoietic drive such as iron deficiency anemia (IDA) (Stoffel et al. 2019). Two missense variants in the coding-region of the *HFE* gene are responsible for the majority of HH cases: the rs1800562 (C282Y) in exon 4, a G to A transition at nucleotide position 845 resulting in a cysteine to tyrosine substitution at amino acid 282, which abrogates HFE protein from reaching the cell surface and interacting with TFRs and hepcidin, and the rs1799945 (H63D) in exon 2, a C to G transition at nucleotide 187 resulting in histidine to aspartic acid substitution at amino acid 63, which reduces the affinity of the altered protein for TfR1 (Barton et al. 2015; Katsarou et al. 2016; Wang et al. 2017). Hence, due to malfunctioning in BMP/SMAD pathway of patients with HFE HH and Hfe − / − mice, the expression of hepcidin is inappropriately decreased and the response of hepcidin to iron intake is diminished (Pantopoulos 2008, 2018).

Various studies investigated the potential role of iron homeostasis in brain disorders and specifically primary headaches, with some reports indicating a link between elevated iron levels in brainstem pain-processing structures and primary headaches. Welch et al. reported enhanced iron levels in the periaqueductal gray matter (PAG), a descending antinociceptive-modulating center, of episodic migraine patients with and without aura and in patients with chronic daily headache, that further elevate with the duration of the disease (Welch et al. 2001). In line with these findings, Kruit et al. showed enhanced iron deposition in multiple central pain mediating-nuclei, predominantly in putamen, globus pallidus, and red nucleus, with higher iron accumulation in migraineurs with longest disease history (Kruit et al. 2009). Conversely, the 9-year follow-up study failed to provide supportive evidence for progressive iron accumulation in deep brain nuclei related to migraine; nevertheless, the authors explained that these findings may be attributed to age-related iron increase, thus age can serve as conflicting factor (Palm-Meinders et al. 2017). Tepper et al. confirmed the association between increased iron deposition in basal ganglia, particularly in globus pallidus, and migraine risk as well as attack frequency and disease duration (Tepper et al. 2012). A more recent study supported the hypothesis of an increased iron deposition in basal ganglia nuclei, especially red nucleus and PAG, in chronic migraine patients (Domínguez et al. 2019). Few studies indicated an association between IDA and migraine incidence, particularly in females (Gür-Özmen and Karahan-Özcan 2015; Tayyebi et al. 2019). Furthermore, a large study in a Norwegian population indicated that phenotypic hemochromatosis, a progressive iron overload disease, and/or *HFE* C282Y/C282Y genotype carriers were both associated with increased headache prevalence among female participants (Hagen et al. 2002). Coexistence of migraine and CH has been documented in some patients and a genetic link for these primary headache disorders has been suggested (Kudrow and Kudrow 1994; D’Amico et al. 1997). Lastly, hereditary haemochromatosis was reported in two cousins with CH, while normalization of iron deposits improved temporarily CH attacks (Stovner et al. 2002). Consequently, if elevated iron levels in the brain pain-modulating areas are implicated in headache pathophysiology, the co-existence of hemochromatosis could potentially have an impact on disease progression or development. Although the more common *HFE* H63D variant rarely causes HH, it is suggested to be a disease risk and progression modifier for several neurological disorders (Kim and Connor 2020). Thus, the *HFE* H63D variant serves as an interesting candidate genetic variant for CH susceptibility.

The aim of the current association study was to examine whether the rs1799945 (H63D) *HFE* variant might confer susceptibility to develop CH and/or diverse disease phenotypes in a cohort of Southeastern European Caucasian (SEC) CH patients and neurologically healthy control subjects.

## Subjects and Methods

### Study Population

A total of 128 unrelated SEC CH patients (95 males and 33 females), aged between 22 to 68 years (mean ± standard deviation: 41.7 ± 10.3 years; 75.8% male) served as CH group. The diagnosis of CH was carried out by experienced neurologists according to the International Classification of Headache Disorders criteria (ICHD-3). The main clinical characteristics of CH cohort and demographic data for the study population are summarized in Table 1. Case subjects were recruited from specialized headache clinics located in Glyfada and Thessaloniki, Greece. Two hundred ninety-four unrelated neurologically healthy SEC control subjects (149 males, 145 females) with no personal and family history of migraine or any other headache disorder, aged between 21 to 85 years (mean ± standard deviation: 57.3 ± 13.0 years; 50.7% male) were recruited from the Neurology Department, University Hospital of Larissa, Greece. Data collected from control subjects included only age and gender. All study subjects were South-eastern European Caucasians. A written informed consent was provided by each study subject. The research was performed in accordance with the principles outlined in the Declaration of Helsinki. The study protocol was reviewed and approved by the appropriate Ethics Committees (Mediterraneo Hospital, Glyfada, Greece, and University Hospital of Larissa).

**Table 1:** Demographic and clinical characteristics of the study population

	**CH patients **(***N*** = 128)		**Controls **(***N*** = 294)
Age (mean ± SD)	41.7 ± 10.3 ranged from 22 to 68 years		57.3 ± 13.0 ranged from 21 to 85 years
Gender (M/F)	95/33		149/145
BMI (mean ± SD)	25.8 ± 4.5 kg/m^2^		-
Age of diagnosis (mean ± SD)	37.1 ± 10.8 ranged from 18 to 65 years		-
Positive family history	57	(44.5%)	-
Type of CH			
ECH	88	(68.8%)	-
CCH	40	(31.2%)	-

### DNA Purification and Genotyping 

Epithelial cells from the oral cavity of the study subjects were collected by sterile buccal swabs. Genomic DNA was extracted from the epithelial cells with the Nucleospin Tissue DNA Mini Kit (Macherey–Nagel GmbH & Co., KG, Düren, Germany) according to the manufacturer’s protocol. DNA concentration in the purified samples was quantified by NanoDrop One Spectrophotometer (Thermo Fischer Scientific), and the samples were stored in −20 °C until screening of the selected *HFE* variant. H63D (rs1799945) genotypes of each participant were determined by the SNP TaqMan qPCR method using the Applied Biosystems QuantStudio 12 K Flex Real-Time PCR System.

### Statistical Analysis

Continuous variables are shown as mean ± standard deviation (SD). Categorical variables are presented as frequencies (*n*) and percentages (%). Chi-square (*χ*^2^) (Pearson or Fischer’s exact when appropriate) tests were used to compare H63D variant frequency distribution between groups and CH subgroups under co-dominant, dominant, over-dominant, recessive genotypic, and allelic inheritance models. Multivariable logistic regression analysis was used to adjust for potential confounding factors including age and gender as covariates, to exclude any bias due to the skewed age distribution and gender ratio between the groups. The crude and adjusted odds ratios (OR) with the corresponding 95% confidence intervals (CI) were calculated to estimate the risk derived from statistically significant differences. Statistical analysis of the data was carried out using version 26.0 of the IBM SPSS Statistics software for Windows. All *p*-values were two-sided, and values less than 0.05 were considered statistically significant. The web-based PS: power and sample size calculation program (v3.1.6), was used for power analysis (https://vbiostatps.app.vumc.org/ps/). With the available sample size and a minor allele frequency (MAF) of 0.14, true odds ratios (OR) below 0.32 or above 2.13 can be detected for CH cohort with 80% power (Dupont and Plummer 1990). Deviation from the Hardy–Weinberg Equilibrium (HWE) in control subjects was assessed with the web-based Online Encyclopedia for Genetic Epidemiology studies software (Rodriguez et al. 2009).

## Results

### Genotype and Allele Frequency Distribution analysis Between Cases and Controls

The genotypic call rate for *HFE* H63D (rs1799945) was 98.8%. Consistency with HWE in the control group was verified for the *HFE* variant (*p* > 0.05). The genotype and allele frequencies of the investigated genetic variant in CH patients and controls are presented in Table 2. Chi-square test and logistic regression analysis with age and gender as covariates showed no statistically significant difference in genotypic and allelic frequency distribution of the *HFE H63D* variant between case and control subjects, in any of the genetic inheritance model tested (*p* > 0.05), possibly indicating an absence of association between H63D variant and susceptibility to CH in the current SEC population. (Table 3).

**Table 2:** Genotypic and allelic frequency distribution of *HFE* H63D variant in cluster headache patients and control subjects

	**CH patients **(***N*** = 128)				**Controls **(***N*** = 294)			
	*Male*	*Female*	*Total*		*Male*	*Female*	*Total*	
***H63D* rs1799945**	*(n* = *91)*	*(n* = *32)*	*(n* = *123)*	*%*	*(n* = *149)*	*(n* = *145)*	*(n* = *294)*	*%*
CC	69	21	90	(73.2)	116	104	220	(74.8)
GC	19	10	29	(23.6)	27	39	66	(22.4)
GG	3	1	4	(3.3)	6	2	8	(2.7)
Missing	4	1	5				-	
C	157	52	209	(85.0)	259	247	506	(86.1)
G	25	12	37	(15.0)	39	43	82	(13.9)

**Table 3:** Frequency distribution analysis in cluster headache patients and control subjects for the *HFE* H63D variant

***HFE*** **H63D genotypes/alleles**	**OR (95%CI)**	***p*-value**	OR (95%CI)*	*p*-value*
CC vs. GG	0.818 (0.240–2.785)	0.751**	1.965 (0.444–8.697)	0.374
CC vs. GC	0.931 (0.564–1.536)	0.780	0.828 (0.455–1.506)	0.536
GC vs. GG	0.879 (0.245–3.152)	1.000**	2.224 (0.454–10.896)	0.324***
CC vs. GC + GG	0.917 (0.569–1.480)	0.724	0.919 (0.520–1.625)	0.772
GG vs. GC + CC	1.202 (0.355–4.067)	0.754**	0.487 (0.111–2.139)	0.341
GC vs. CC + GG	1.066 (0.647–1.754)	0.802	1.237 (0.684–2.236)	0.482
C vs. G	0.915 (0.601–1.394)	0.680		

*OR* odds ratio, *CI* confidence interval

^*^Adjusted for gender and age

^**^Fisher’s exact test 2-sided

^***^Hosmer–Lemeshow test *p*-value < 0.05

### Subgroup Analysis According to CH Clinical Phenotypes

In order to investigate the association between *HFE* H63D variant and diverse CH phenotypes, patients were stratified into subgroups according to the form of CH (episodic/chronic) and the frequency of attacks. The CH group included 85 (69.1%) patients diagnosed with episodic CH form and 38 (30.9%) chronic CH patients. A statistically significant correlation between the *HFE* H63D and CH type was revealed. As listed in Table 4, the frequencies of both CC genotype and C allele in CH cohort were significantly higher in patients with episodic compared to chronic CH patients (OR [95% CI] 2.428 [1.055–5.584], *p* = 0.034 dominant genotypic model and OR [95% CI] 2.161 [1.059–4.410], *p* = 0.032 allelic model). The difference remained significant after adjustment for age and gender (OR [95% CI] 2.443 [1.051–5.679], *p* = 0.038 dominant model). Consequently, CH patients homozygous (CC) for the H63D C allele may be at lower risk for disease chronification compared to carriers of the H63D G allele. No statistically significant association of the *HFE* H63D variant with the frequency of CH attack occurrence was detected in the current SEC CH cohort (data not shown).

**Table 4:** Frequency distribution analysis in episodic and chronic cluster headache (CH) patients for the *HFE* H63D variant

**Genotypes/alleles**	**Episodic CH (***N* **= 85)**		**Chronic CH (***N* **= 38)**		**OR (95%CI)**	***p*-value**	OR (95%CI)*	*p*-value*
	***n***	**(%)**	***n***	**(%)**				
CC	67	(78.8)	23	(60.5)	1.0 (reference)	-	-	-
GC	16	(18.8)	13	(34.2)	2.367 (0.990–5.659)	**0.049**	2.322 (0.963–5.599)	0.061
GG	2	(2.4)	2	(5.3)	2.913 (0.388–21.880)	0.287**	3.170 (0.402–24.989)	0.273
GC + GG	18	(21.2)	15	(39.5)	2.428 (1.055–5.584)	**0.034**	2.443 (1.051–5.679)	**0.038**
GG	2	(2.4)	2	(5.3)	1.0 (reference)	-	-	-
GC	16	(18.8)	13	(34.2)	1.231 (0.152–9.972)	1.000**	0.362 (0.033–4.031)	0.409
GC + CC	83	(97.6)	36	(94.7)	0.434 (0.059–3.200)	0.587**	0.372 (0.049–2.842)	0.341
GC	16	(18.8)	13	(34.2)	1.0 (reference)	-	-	-
CC + GG	69	(81.2)	25	(65.8)	0.446 (0.188–1.057)	0.063	0.453 (0.189–1.087)	0.076
C	150	(88.8)	59	(77.6)	1.0 (reference)	-	-	-
G	20	(11.8)	17	(22.4)	2.161 (1.059–4.410)	**0.032**	-	-

*OR* odds ratio, *CI* confidence interval

^*^Adjusted for gender and age

^**^Fisher’s exact test 2-sided

## Discussion

The current study examined the association of H63D (rs1799945) variant in the *HFE* gene with the susceptibility to develop CH and diverse clinical phenotypes, in a SEC case–control population. Genotypic and allelic frequency distributions of the H63D genetic variant were similar between patients and control subjects, indicating no association between this *HFE* variant and CH risk susceptibility in SECs. Nonetheless, the stratified genotypic and allelic analyses according to CH subgroups (episodic and chronic CH) showed that the variant G allele was significantly more prevalent in chronic compared to episodic CH patients, conferring an increased risk for disease chronification for the patients carrying the variant G allele. Thus, although it is unlikely that *HFE* genetic variation contributes considerably to CH genetic susceptibility, a potential role for *HFE* as a disease modifier gene may be regarded.

The possible molecular mechanisms interpreting the findings of the current study are not clear. As mentioned, various studies suggested a correlation between increased iron deposits in brainstem pain-processing structures and primary headaches. The HFE protein is expressed in the brain along with TfR; thus, variants in the *HFE* gene may influence brain iron uptake. *HFE* variants are related with enhanced iron accumulation (Bell et al. 2021); therefore, H63D variant might regulate iron deposition in brain pain-modulating centers and contribute to brain iron overload and increased oxidative stress states. Iron overload may alter the threshold for headache triggering, rendering carriers of *HFE* variants more susceptible to environmental challenges and/or other genetic modifiers (Nandar and Connor 2011; Nandar et al. 2013). Moreover, the presence of *HFE* variant genotypes may confer susceptibility for CH even in the absence of enhanced iron deposits. Lastly, a correlation between iron-deficiency anemia (IDA) and the incidence of migraine was suggested (Gür-Özmen and Karahan-Özcan 2015; Tayyebi et al. 2019). To maximize iron absorption in IDA, hepcidin levels are usually repressed (Girelli et al. 2016; Dewan et al. 2019). Since the HFE protein is involved in the regulation of hepcidin expression, the underlying biological mechanism may be associated with defective hepcidin pathway.

Only one previously reported study by Rainero et al. investigated the impact of C282Y and H63D *HFE* missense polymorphisms on CH occurrence and clinical features, in an Italian cohort of 109 CH patients and 211 age-matched and geographically matched healthy controls. They observed no C282Y variant in the Northern Italian population of the cohort, whereas, similarly to the current study, they concluded that the examined *HFE* variants are not significantly associated with CH. On the contrary, they found no significant difference between episodic and chronic CH patients. The lack of statistically significant difference may be attributed to the small number of chronic CH patients included in the Italian CH cohort (14 chronic CH patients) and the absence of D63D genotype carriers in the chronic CH subgroup. Lastly, they observed a later age of disease onset in the four D63D homozygous patients, indicative of an involvement of *HFE* gene in disease modification (Rainero et al. 2005). An association study in an Italian population of 256 migraine patients and 237 healthy age-, sex-, and ethnicity-matched controls showed that patients with the H63D GG (D63D) genotype presented significantly later age of disease onset and increased frequency of migraine attacks, concluding that the *HFE* H63D variant may be a modifying genetic factor in migraine (Rainero et al. 2007).

Certain limitations of the current study should be acknowledged. Firstly, the relatively small sample size may not be sufficient to reveal small effects of the examined genetic variants in CH. Secondly, the study population was enrolled from a particular geographical area and does not consist of ethnically or racially different populations to avoid population selection bias; thus, it should be considered when interpreting the results in other populations. Other potential bias might be the difference in gender ratio and age distribution between case and control subjects, although an adjustment in statistical analysis of the results was performed. Finally, the study did not evaluate confounding factors such as gene–gene or gene-environment interactions which may possibly influence CH phenotypes.

## Conclusion

Even though CH is regarded as a multifactorial primary headache disorder with both genetic and environmental inputs, the phenotypic variability defined by genetic variation is still uncertain. The results of the current study did not provide supportive evidence for significant association between the investigated *HFE* gene variant and CH susceptibility in the SEC population; hence, *HFE* gene might not be considered as a genetic risk factor for CH. Nevertheless, a correlation between H63D *HFE* variant and disease progression could be perceived; thus, in accordance with previous studies in primary headaches, *HFE* may serve as a candidate disease modifier gene. More, larger scaled studies in different populations, considering gene–gene and gene-environment interactions as well as additional iron metabolism regulating genes and biochemical parameters should be followed to elucidate the precise role of HFE and iron homeostasis in CH pathophysiology and their possible involvement in CH genetic susceptibility and diverse phenotypic characteristics. Detecting the underlying genetic components may potentially unravel the molecular pathways implicated in disease pathophysiology and facilitate the development of novel therapeutic targets.

## Data Availability

Available upon reasonable request.
